# Health care efficiency and climate change implications linked to reproductive health in developing countries

**DOI:** 10.1186/1472-6963-12-S1-O7

**Published:** 2012-11-21

**Authors:** David Baguma, Jamal Hisham Hashim, Syed Mohamed Aljunid

**Affiliations:** 1 United Nations University International Institute for Global Health (UNU-IIGH), Kuala Lumpur Malaysia

## Introduction

Health care services for pregnant women and children are partially influenced by externalities such as climate change and environmental hazard health risks i.e., the vulnerabilities confounded within food insecurity and extreme weather events (floods) in drought prone geographical regions. The unhygienic conditions due to lack of adequate safe water and sanitation also compromised their health status. In this paper, we further attempt to examine the possible implication of climate change hazards on pregnant women and children exploring efficiencies within health care systems.

## Materials and methods

The data on population growth rates between 1650 and 2008, the reproductive health literature (use of contraceptive and pills between 1993 and 2007), and climate change effects (temperature variation and changes in diseases reported such as diarrhoea or vector-borne diseases transmitted by malaria and dengue) were collected on countries: Bangladesh, Malaysia and Uganda. The data were reviewed and descriptive analyses used to give an overview on the possible implications of climate change to health care efficiency in South Asia and Sub-Saharan Africa.

## Results and discussion

The findings show that temperature variations are associated with increase in numbers of mosquitoes, which could link malaria related deaths to changes in climate scenarios. This study’s findings also indicate that efficiency in health care is affected by housing condition, cultural beliefs, levels of income and education, and water and sanitation. The number of diarrhoea related cases for children (< 5 years) increased with periods when rainfall was high, which links climate change to water-borne diseases. This study supports findings which found that natural immunity in women is suppressed especially during pregnancy making them vulnerability to water-borne illness (such as caused by unhygienic water sources), which is exacerbated by inefficient health care systems and services.

## Conclusion

Health care efficiency could be enhanced by improvements in capacity building e.g., training in antenatal and paediatric care, or adoption of technologies that improve information flow in health facilities e.g., Casemix systems for documentation. To improve health care efficiency, we also recommend emphasis on effective interventions for the prevention and control of diseases at all level of health care structures (i.e., environmental health services), foster climate change adaptation and mitigation measures.

